# A teaching tool for tic disorders: using “disinhibition” to unify the spectrum

**DOI:** 10.3389/fpsyt.2025.1577983

**Published:** 2025-06-09

**Authors:** Samuel H. Zinner

**Affiliations:** ^1^ Department of Pediatrics, University of Washington, Seattle, WA, United States; ^2^ Division of Developmental Medicine, Seattle Children’s Hospital, Seattle, WA, United States

**Keywords:** Tourette syndrome, chronic tic disorders (CTD), disinhibition, medical home, primary care advantage, meaningful learning

## Abstract

Chronic Tic Disorders (CTDs) including Tourette’s Disorder are common pediatric conditions that, like many other mental health conditions, are under-recognized and under-managed in the primary care setting. Pediatric Primary Care Providers (PCPs) often feel undertrained in mental health evaluation and management, but the Medical Home partnership between PCPs and their patients and families usually serves as the most available opportunity for timely and comprehensive assessment and care coordination of mental health concerns; PCPs and families may feel confused, surprised, and bewildered by the wide range in the clinical presentations of tics and their seemingly dissimilar coexisting conditions that appear to blur the margins between mental health and neurology. This article endeavors to teach PCPs - who can thereby teach families of children with CTDs - a general clear and understandable principle that bridges mental and behavioral health with neurophysiology. This principle is one of “disinhibition,” which is believed to underpin tic behaviors and a variety of symptoms of frequent coexisting conditions. The teaching approach set out in this article is schematic and takes advantage of active learning methods. Once understood, families and PCPs can then use their knowledge of disinhibition to help prioritize concerns and engage with greater purpose and direction in management of symptoms, anticipation of possible symptom evolution, and to advocate more persuasively for appropriate educational or related services when indicated.

## Introduction

Tic disorders are common and routinely encountered in the primary care Medical Home setting by pediatric primary care providers (PCPs), with up to 1 in 5 children experiencing tics at some point. Less commonly, tics become persistent (lasting a year or longer), and are then identified as Tourette’s Disorder (motor *and* vocal tics) or as a Persistent Tic Disorder (motor *or* vocal) (1), with tics often continuing into adolescence and beyond. For this discussion, Tourette’s Disorder and other persistent tic disorders will be grouped and referred to as Chronic Tic Disorders (CTDs).

It is very common for individuals with CTDs also to have related behavioral, learning, and/or emotional mental health coexisting conditions that can emerge during early childhood or beyond (2). Because of these coexisting conditions and their psychosocial impacts, tic disorders, while classified as *movement* disorders, are also *mental health* conditions and can be grouped among the “neuropsychiatric” disorders. In fact, during preparation of the 5^th^ edition of the Diagnostic and Statistical Manual of Mental Disorders (DSM-5), controversy emerged over whether to move tic disorders, including Tourette’s Disorder, to a proposed new section called “Anxiety and Obsessive-Compulsive Disorders” (3).

Individuals with CTDs contribute to the pool of the 1 in 5 children and adolescents with diagnosable mental health disorders toward whom the American Academy of Pediatrics’ Mental Health Initiatives have been directed (4). About half of all visits to a pediatric PCP involve concerns related to mental health considerations that encompass behavioral, psychosocial, emotional and/or educational concerns (5). Primary care providers are often the first and most enduring healthcare providers in a child’s life, so they are critically important in the recognition and treatment of CTDs and coexisting conditions (6), but often have insufficient background and training in this area, resulting in underdiagnosis and underserving of patients and families (7, 8). Only 20% of children and adolescents with a diagnosable mental health condition who seek treatment receive needed services (9), a deficiency to which children with CTDs are particularly vulnerable (10). Despite decades-long ongoing efforts to strengthen primary care competence and confidence in this area, identification and management of CTDs is often relegated to healthcare specialists outside of primary care, who are often in short supply, contributing to access barriers (11, 12) and delayed diagnosis (13, 14).

Mental health care is central to good primary medical care, as mental and physical health are interdependent (15), but the majority of recently-trained pediatricians continue to report not feeling competent in providing mental health treatment (16). This perpetuates underdiagnosis and undermanagement and sets the stage for perpetuating the ongoing discomfort among faculty in pediatric training programs in teaching behavioral and mental health primary care (17).

Early identification, monitoring, prevention, and management of tics and their coexisting conditions is imperative in setting a lifelong optimal course to build and maintain efficacy and self-esteem (18), as most adult mental health disorders, including anxiety and depression, originate in childhood and grow increasingly difficult to manage, but may be lessened or prevented by early identification and management (15). And, PCPs who can communicate complicated medical information about CTDs using simple and effective teaching ideas can help patients and their families build more positive attitudes about tics and their association conditions (19). Failure to do so may contribute to decline in quality of life and lead to poorer psychosocial functioning (20). Given this lifelong trajectory, competent diagnosis and management of CTDs within a pediatric primary care Medical Home is a critically important goal, and a clear and understandable strategy to help PCPs and families better understand and manage tics and their related conditions is imperative.

## Medical Home and the mental health imperative

The Medical Home model was introduced by the American Academy of Pediatrics in 1967 to promote healthcare that partners families with PCPs as the go-to contact, priorities accessibility, continuity, coordinated care, and cultural effectiveness (21), and emphasizes comprehensive care that incorporates mental health services as a central tenet of its role (22).

Pediatric PCPs have a “Primary Care Advantage” in recognizing and managing mental health conditions; this advantage is intrinsic to their role in monitoring a child’s developmental and behavioral achievement, delivered in the continuous therapeutic relationships of the Medical Home. This mental health directive is not new; its roots were formally planted in 1978 with the publication of the *Future of Pediatric Education Task Force Report* and updated 2000 (23, 24). A number of subsequent initiatives have been published, and a mandate requiring a 1-month block in developmental-behavioral pediatrics during general pediatrics residency training has been implemented (25), further emphasizing this critical professional training and service need in pediatric primary care (17). In response, strategies proposed, designed, and/or implemented to help enhance the primary care advantage include improving fundamental communication skills to engage youth and families regarding social and mental health problems (4), and integrated behavioral health care models that join primary and mental health subspecialist providers in collaborative care (5, 26–28).

## Disinhibition as a unifying principle in chronic tic disorders and related conditions

A bridge to helping pediatric PCPs better understand connections between physical and mental health aspects in tic disorders - knowledge which can then be used clinically in communicating with families of their patients - can be found with the phenomenon of ‘disinhibition’. Tics and their most frequent coexisting conditions (including attention-deficit disorders, obsessions/compulsions, and anxiety disorders) may share disinhibition as a common ‘brain-based’ (neurological and mental health) vulnerability. The general idea behind disinhibition is this: information that is warehoused in the brain and ordinarily blocked or filtered from awareness until summoned instead ‘leaks’ out into awareness inappropriately. It’s believed that disinhibition contributes to a range of possible symptoms (tics and others) (29–31), each of which can be conceptualized as having 3 phases: Before, During, and After the occurrence of the behavior pattern of interest (tics and other frequent coexisting behaviors). Through the widely familiar and often misunderstood psychological mechanism of “reinforcement”, Before, During, and After symptoms can be either strengthened or reduced. By understanding disinhibition and reinforcement as shared across a range of symptoms, clinicians and families can better identify, monitor, and address symptoms of concern, including tics, ADHD, OCD, anxiety and others, while also enhancing a child’s own identity and agency.

Reaching a shared understanding of disinhibition requires clear and simple communication between the PCP and family and is key to making the meaningful connections needed to effectively understand and use medical information. Often in primary care, communication concerning neurodevelopment falls short (32, 33). Within communication styles, the ability of a clinician to provide good-quality, tailored and understandable information with children and families is among the most important clinical features in promoting connection between the provider and family and, thereby, growth and behavior change in a child’s mental health (34). Conversely, poor quality information about a condition inappropriately tailored to the child and family’s health literacy needs, as well as limitations of available time during the clinical encounter, are among the most consequential of barriers (33). Because over one-third of US adults have limited health literacy (i.e., the degree to which they can obtain, process, and understand basic health information and services needed to make appropriate health decisions) (35), clear communication (avoiding jargon and excessive information, and confirming patients’ understanding of what was discussed) is crucial (36).

The necessity for clear and accurate communication in health care delivery is keenly relevant to CTDs, where misinformation (via internet, entertainment media, and other sources) (37) and misunderstanding (38) are rampant, and where shame or parent guilt are frequent associations (39, 40). Further, because the vast majority of individuals identified with CTDs have or will later develop coexisting conditions, which may include ADHD, obsessions and compulsions, anxiety disorders, anger dysregulation, autism or related social pragmatic communication interferences, among others (41), a family’s ability to grasp that diverse symptoms - both current and/or future - can share an underlying easy-to-understand origin is of practical social and clinical relevance toward symptom monitoring, prevention and management, finding common ground, repairing blame, and educating others about this spectrum condition (42).

## Teaching and learning principles: make it organized and meaningful

To help clinicians teach families about tic and related disorders, we can take advantage of evidence-based principles within the cognitive science of memory and learning; research findings demonstrate that we learn best when new information is meaningful to us, such as how the information relates to, or builds on, something we already understand or care about (43). A variety of evidence-based teaching modalities tap into these principles and can be applied in teaching both medical professionals and parents. Additionally, when applied during clinical encounters concerning disinhibition, meaningful learning can help clinicians and parents have a more usable understanding of the child’s current and future emerging tic and related behaviors.

Features of meaningful learning modalities should begin with a *common factors* communication approach that demonstrates empathy and understanding of the specific symptoms and vulnerabilities as viewed by the child and family, and conveys humility and commitment in partnering with the family in order to collaborate on building a workable plan (4).

Beyond this point of orientation, additional teaching methods that strengthen meaningful learning can emerge: Begin with a theme that captures the *big picture* that is built on a shared understanding with the learner of the concern, and that factors into their personal beliefs or fears; Be conscientious of complexity and aim to communicate with *clarity and simplicity*; Use *dual coding* (two or more methods) that include both verbal and visual representations, to illustrate and integrate understanding of a concept, and use specific, *concrete examples* to help the learner understand an abstract principle; Apply *scaffolding* to elaborate on an idea that a learner understands; Speak using conversational and *patient-friendly words*, not jargon (44); Use *analogies or metaphors* to liken a new and complicated point with one that’s already familiar (45); *Repeat key points* and *check in* with learners to see that everything is clear; and, Assist learners by *helping them to ask questions* and, when needed, by anticipating or structuring – and then answering – questions (4, 46–48). These italicized points will be referred to in the discussion below.

As a Developmental-Behavioral Pediatrician, I generally provide consultation on patients at the request of a PCP. In the discussion below, I introduce a specific set of teaching methods I’ve developed to educate families and clinicians about tic disorders and their coexisting conditions, with an intention of enhancing the effectiveness of primary care management in the Medical Home relationship. The focus of the discussion below will be on tics, either as the principle concern or as an additional presenting or emerging concern during the clinical encounter.

I begin by asking each participant their concerns and their understanding of tic disorders (*common factors*). Related concerns that either indicate or have been established as coexisting difficulties may also be raised. When a diagnosis of a CTD is suspected or established, I begin by saying that I do not view tics as a diagnosis; instead, I see tics as symptoms of a broader diagnosis. This other diagnosis, I explain, does not have an official name, so that I’ll name it myself: “Leaky Brain Filter” (*patient-friendly words*), and that the filter is meant to block, or “inhibit,” unneeded brain information. Leaky Brain Filter can cause lots of symptoms, including tics. This construct sets the stage for thinking more broadly and dimensionally about mental health, shifting from rigid categories (49, 50).

I take a few minutes to discuss what is believed to cause tics, how tics may change over time, and why we may see a variety of related other developmental and behavioral patterns or conditions (*the big picture*). I share the somewhat disarming quotation attributed to physicist Emerson Pugh, “*If the human brain were so simple that we could understand it, we would be so simple that we couldn’t*” and I let families know that what I am about to share is an oversimplification (51), but that it captures a key aspect (*communicate with simplicity*) and will be helpful to us, clinicians and families jointly, in understanding the child’s behaviors and in charting a management path.

**Figure 1:**
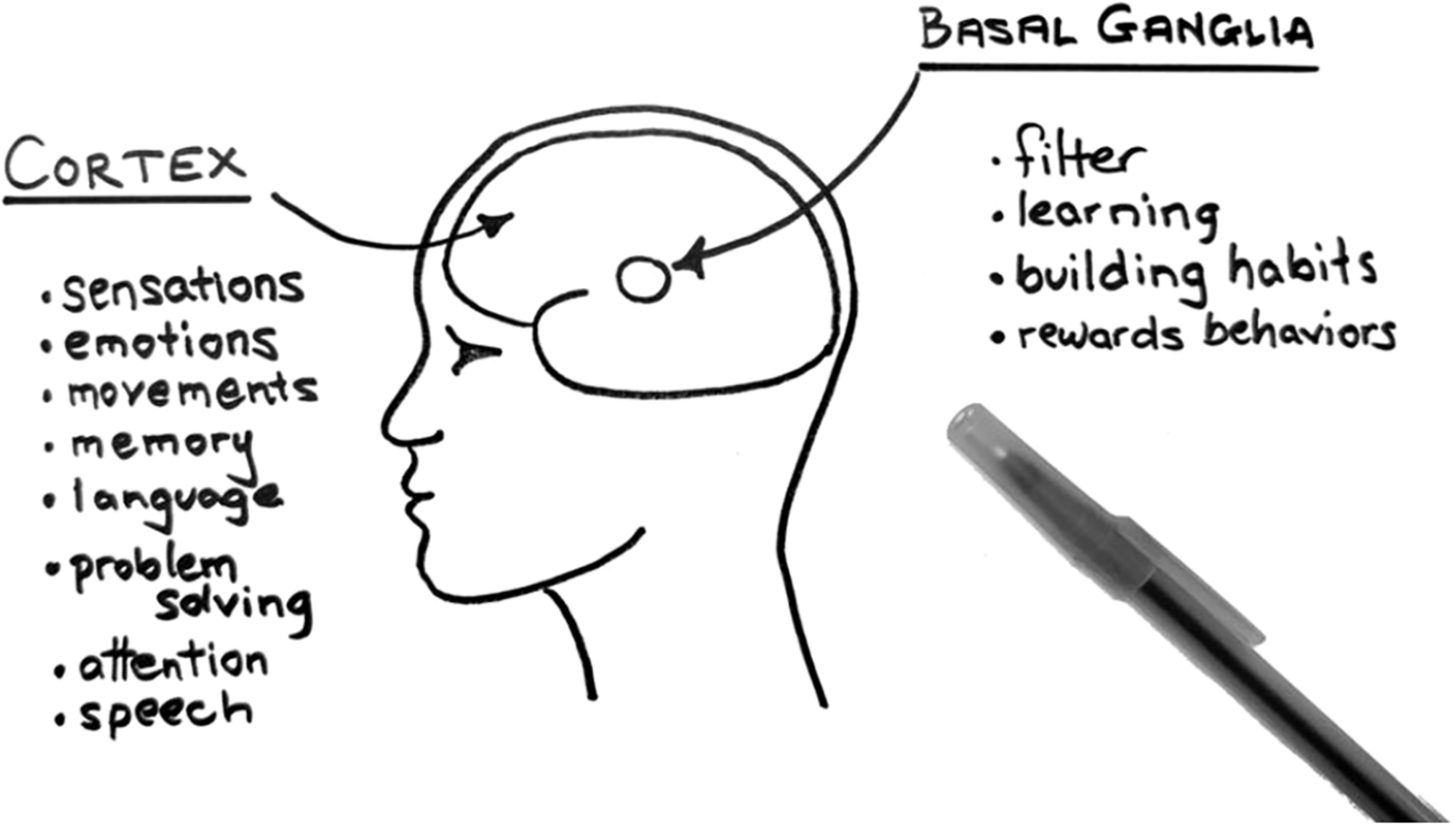
Schematic representation of human brain as “outside” and “inside” parts with key functions.

Anatomy and Physiology: I draw a simplified picture of the outer outline of the human brain in profile (*dual coding*) with a small circle within to represent the basal ganglia (see **Figure 1**). I share that the brain is made up of many parts, but we can think of it as a ball with 2 parts: the outside squiggly part (“cortex”, which is Latin for “bark” like the outside of the tree [*analogy*]) and the inside part below the outside (“subcortex”, where “sub” is Latin for “below”, or “below the bark”, the center of the ball).

I share that the brain is made up of many kinds of brain cells but that for our discussion, we’ll treat them as though they are all the same. I note that there are nearly 100 billion brain cells, about the same number as stars in our Milky Way Galaxy (*analogy*), each cell nearly helpless, useless on its own; rather, the brain works as a network, with cells talking with each other. To do so, each cell has about 1,000 “arms” or “tubes” (*patient-friendly words*) called axons that connect to other brain cells, making up perhaps 100 trillion connections, too large a number to grasp. At all times, cells are talking with each other in this network (*repeating the key point “network”*) by “spitting out and slurping up” chemicals (“neurotransmitters”) and electricity. There are at least 100 different kinds of chemicals stored among brain cells, including some probably familiar by name (such as dopamine, epinephrine – also called adrenaline, and serotonin). Different combinations and quantities of these chemicals are repeatedly being traded across the 100 trillion connections between cells, along with electrical impulses. I *check in* for understanding.

Filter: Returning to the simplified picture of the brain, I point out that the cortex stores a variety of categories of brain information, including movement, sensations, emotions, language, memories, problem solving, social rules, pain and temperature, and other functions. The cortex is always sending out and receiving combinations of chemical and electrical information to nearby and distant cells, including noise signals that should be blocked out unless it’s needed here and now. So, cells also send axons from the cortex to the basal ganglia, a gumball-sized filter in the subcortex with many functions, key among them filtering (or “inhibiting”, “blocking”, “gatekeeping”, or “gating”) the noise. The filter opens (“disinhibits”) when signals are needed and stays closed to these signals when they are not. To illustrate this point, I ask the parent to notice the sensation of the floor on the soles of their feet. I point out that their brain had been “asking permission” to notice this sensation before I mentioned this, but as soon as I had asked the parent to notice, the filter then opened some gates to notice a combination of signals, including language (“feet”), location (“floor”), sensation (pressure or temperature on their feet), etc.

I continue by relating that all that we know and learn is stored in “packages” in the brain connected within the networks, and that most information is made up of combinations of the different categories stored in the cortex. So, for example, what we observe as a movement (like a tic) is actually made up of many different categories of information or experiences, including sensations, movements, emotions, and memories, among others.

Behaviors as packages of neurological categories: At this point, I introduce the analogy of tics to eyeblinks (*concrete example*), noting that we all blink several times each minute without noticing it, unless we turn our attention to our eyes. If we try not to blink, we notice a sensation. I will ask the child (or parent) to describe what they notice when they try not to blink, such as when playing “the staring contest” (last person to blink wins). Usually, the child can describe an unpleasant sensation that builds around their eyes, like a burn, tickle, itch, or a pressure, which grows too intense to resist. The child blinks, then feels relief. I point out that tics, like blinking, have 3 phases: Before (unpleasant sensation), During (blinking movement), and After (emotional relief) the blink. If a child’s tic patterns include blinking (as is often true), I ask the child whether their blinking tics are similar to the staring contest analogy (it often is).

Disinhibition: Having now built a schema to organize an understanding of brain anatomy and physiology, I repeat that our skills and knowledge are combined and stored as “packages” of movements and sensations and emotions that travel together through the brain and reach the filter where, in essence, the package of information knocks on the door of the filter for permission to enter (i.e., to be noticed or perceived or activated). Most often, this information or action is not needed, and so it is inhibited, or filtered, from our awareness.

However, in people with Leaky Brain Filter, there are holes in this filter. I emphasize that the child’s brain works well and is not broken, and I’m careful to remind that I’m simplifying and distorting - the filter does not have true holes - to help us understand tics and their related conditions. I *check in* for understanding.

I reintroduce the example of blinking, mentioning it as perhaps the most common tic, and often the very first tic to appear. Movements and other behaviors that happen frequently, like blinking, show up more frequently at the filter. When there is a “leak”, the combined package (sensation, movement, emotion) filters through instead of getting blocked, and reaches into awareness, driving the child to blink, sometimes strongly and often several times in a row, until the child feels relief.

Reinforcement: Next, tics are strengthened through a psychological process called “reinforcement”. Most often, parents are familiar with the terms “positive reinforcement” and “negative reinforcement”. However, I warn, they probably mistake “negative reinforcement” to mean punishment. I describe both positive and negative reinforcement as resulting in something desired, and I write down the following sequence:

***Before > During > After***

I share that usually the term “ABC” (to mean “Antecedent”, “Behavior”, and “Consequence”) is used, but that I find the words Before - - During - - After easier to understand. In this sequence, “During” refers to a behavior, such as a tic. What happens “Before” and/or “After” the behavior can make it more likely for the tic to become more routine and automatic (reinforced). With “positive reinforcement” the focus is on After. With “negative reinforcement” the focus is on Before. “Positive” refers to adding something. “Negative” refers to removing something. With positive reinforcement, a behavior (During) is done and a reward is added (After). With negative reinforcement, something unpleasant exists Before the behavior, and doing the behavior removes the unpleasant thing. For example, an unpleasant sensation, often called an “urge”, emerges (i.e., “Before”) that drives a tic behavior (i.e., “During”) and the urge then resolves with a sense of relief; the removal of unpleasantness and the relief that follows send signals to the filter and to other areas of the brain, teaching the brain, “This tic behavior works!” Because tics repeat, the brain continues to see and experience the pattern again and again, and increasingly strengthens (reinforces) the network of cells in the brain (perhaps by building additional axons to the “package” of connecting cells), making the behavior easier to happen again. Tics are sometimes also positively reinforced; for example, a child whose tic severity increases *During* moments of distress, such as when battling with a sibling over the television remote control, may be rewarded with the remote by a sympathetic parent who trots the hapless sibling off to a time-out (“After”). Again, “This tic behavior works!” (52).

Branching out to coexisting conditions: Having now introduced the models of Leaky Brain Filter paired with the Before > During > After sequence of reinforcement, I use these models as a *scaffold* to relate tic disorders to their coexisting conditions. In most cases, parents will have noticed concerns in their child’s behaviors or learning well before their clinical appointment, and many children will already have been diagnosed with related conditions, such as ADHD or an anxiety disorder. Parents may also have seen the “Iceberg Illustration” of Tourette syndrome, another example of *dual coding* that shows tics at “just the tip of the iceberg”, and with a wide variety of related coexisting conditions making up the rest of the iceberg below the “water’s” surface (https://tourette.org/resource/iceberg-illustration-poster).

The **Table 1** below features symptoms of some of the common coexisting conditions seen in individuals with CTDs. Each symptom can be viewed as an example of a hardwired behavior pattern (each, just like tics, made up of multiple categories of brain information) stored, ready, and available to be perceived and activated when needed. Like tics, each coexisting symptom is frequently “asking the filter’s permission” to be noticed, and each, again like tics, has a Before > During > After sequence that gets reinforced if the full sequence is successfully completed. It can be helpful during the clinical encounter to relate the patient’s own symptoms with the examples presented in the Table to help the child and parents tie these conditions together.

**Table 1:** Coexisting conditions and their “Before > During > After” reinforcement features.

Symptom	Before	During	After
Tics	*Sensation*	*Patterned movement*	*Relief*
Obsessions & Compulsions	*Unwanted thought*	*Patterned behavior*	*Relief*
Impulsive behavior	*Sense of emergency*	*Leap into action*	*Relief (and regret)*
Distractibility	*Boredom or frustration*	*Interrupted attention*	*Mental interest*
Anxiety	*Fearful notion*	*Mental attention*	*Relief/Safety*
Rage Attack	*Perceived threat*	*Aggression*	*Relief (and regret)*

Monitoring and symptom management: It’s beyond the scope of this article to fully discuss evaluation and management, but an instrumental point bears mentioning: the principle of Before > During > After can be applied not only to monitoring, but also to treatment, of tics and related coexisting conditions. Clinicians and parents (and patients) can strengthen their vigilance to the emergence or exacerbation of symptoms. When symptoms are identified, specific cognitive-behavioral treatments can be applied that exploit and reshape the very reinforcers of the offending symptoms, thereby reducing or eliminating them.

## Concluding remarks

Chronic tic disorders are among the most common neuropsychiatric conditions in childhood and adolescence. Pediatric PCPs can help meet the clinical demand for diagnosis and management of CTDs and their coexisting conditions through understanding and effective teaching of a shared neurological mechanism of disinhibition and behavioral principles of reinforcement. By so doing, families can unify the wide range of tic and associated behaviors identified diagnostically under a single organizing idea of Before, During, and After. Understanding the mechanism and principles is very useful to PCPs and families in the clinical identification, management, and anticipatory guidance of tics and coexisting conditions, and can be easily learned and taught using strategies of meaningful learning. Because physical and mental health are intertwined, building this capacity through a clear and simple discussion mechanism within the Medical Home pediatric primary care partnership may offer earlier and more effective identification and management of tic and related mental health conditions and reduced burden on the demand for subspecialists, and may help to contribute to better general overall health and enduring wellness.

## Data Availability

The original contributions presented in the study are included in the article/supplementary material. Further inquiries can be directed to the corresponding author.
